# Exceptional Responder to Immunotherapy: A Rare Case of Post-HSCT DLBCL Relapse Responding to Nivolumab

**Published:** 2019-04-01

**Authors:** Irappa Madabhavi, Sandeep KS, Malay Sarkar, Mitul Modi

**Affiliations:** 1 Department of Medical and Pediatric Oncology, Kerudi Cancer Hospital, Bagalkot, Karnataka, India; 2 Department of Radiotherapy, Kerudi Cancer Hospital, Bagalkot, Karnataka, India; 3 Department of Pulmonary Medicine, Indira Gandhi Medical College, Shimla, Himachal Pradesh, India; 4 Department of Pathology, Gujarat Cancer Research Institute, Ahmedabad, Gujarat, India

**Keywords:** Immunotherapy, Nivolumab, Diffuse large B-cell lymphoma (DLBCL), Chemotherapy, Adverse reactions

## Abstract

Immunotherapy is the treatment that either boosts the patient’s immune system or uses human-made versions of the normal parts of the immune system to kill lymphoma cells or slow their growth.

A forty-eight-year-old lady with neck nodes, axillary nodes, weight loss and fever diagnosed to have Diffuse Large B-Cell Lymphoma (DLBCL) in December 2009 was treated with 6 cycles of R-CHOP, and her treatment was completed in May 2010. After 2 years in July 2012, the patient developed similar symptoms and received salvage chemotherapy with R-DHAP, and her treatment was completed in January 2013. After one and a half years, in August 2014, the patient again had relapsed DLBCL. She was treated with R-ICE 4-cycles and rendered disease-free following allogeneic HSCT in June 2015. But in December 2016, the patient again developed isolated axillary lymphadenopathy and relapsed DLBCL was confirmed by HPR and IHC. This time, the patient was unwilling to go on chemotherapy, but after counselling about the new drug, Nivolumab, she became convinced, and her treatment was started with 3mg per kg every 2 weeks. After 4 cycles, she had a complete response and is now being treated with the same treatment without any symptoms of the disease or any adverse drug reactions. Nivolumab was well tolerated and exhibited antitumor activity in extensively pretreated patients with relapsed or refractory B- cell lymphomas. Additional studies are ongoing to learn more about the use of Nivolumab in these diseases.

## Introduction

 Diffuse large B-cell lymphoma (DLBCL) is the most common type of non-Hodgkin lymphoma. Approximately 60% of DLBCL patients are cured using standard chemotherapy, including monoclonal anti-CD20 antibody (rituximab), cyclophosphamide, doxorubicin, vincristine, and prednisone (R-CHOP). However, 30–40% of DLBCL patients will develop relapse or have refractory disease that cannot be cured with the standard R-CHOP therapy, indicating the need for more effective therapies for these subsets of patients. 

The development of rituximab was an early step in the application of immunotherapy for the treatment of lymphoma, as it was the first monoclonal antibody approved by the US-FDA for the patients with advanced stage or relapsed low-grade non-Hodgkin lymphoma in 1997. More recently, a number of innovative immunotherapy approaches have shown promising results in patients with relapsed or refractory DLBCL → numerous ongoing clinical trials. 

Immune checkpoint blockade has promising potential in DLBCL therapy. A subgroup of patients with advanced cancers may respond to single-agent immune checkpoint blockade^1^. Promising immunotherapy approaches such as chimeric antigen receptor (CAR) T-cell therapy and therapeutic blockade of immune checkpoints, in particular cytotoxic T lymphocyte-associated protein 4 (CTLA4) and programmed cell death protein 1 pathway (PD-1/PD-L1), have boosted the development of new therapeutic regimens for patients with relapse/refractory DLBCL. Immune blockade of the PD-1/PD-L1 interaction by monoclonal antibodies can restore the antitumor activity of cytotoxic T-cells. Early clinical trials using two anti-PD-1 antibodies (nivolumab and pembrolizumab), and three anti-PD-L1 antibodies (avelumab, durvalumab, and atezolizumab) have shown great promise.

## Case presentation

 A 48-year-old lady, non-smoker and non-alcoholic by habits, presented with a 1-month history of neck nodes, axillary nodes, fever, and weight loss. On examination, there was cervical, axillary and inguinal lympadenopathy, moderate splenomegaly, and moderate hepatomegaly. Her routine investigations revealed hemoglobin of 11.5gm/dl, total count of 9600/cu mm and platelet count of 326000/cu.mm. Erythrocyte sedimentation rate (ESR) was 70mm, uric acid was 6.8mg/dl, creatinine was 0.7mg/dl, potassium was 4.2mg/dl, calcium was 9.5 mg /dl, and lactate dehydrogenase was 150IU/L. Serology for human immunodeficiency virus, hepatitis B and C viruses were negative. Positron Emission Tomography-Computed Tomography scan (PET-CT) showed increased Standard Uptake Value (SUV) and hypermetabolic areas in bilateral cervical, axillary, inguinal, mediastinal, para-aortic and iliac lymph node regions, liver, spleen, and vertebral bodies. Bone marrow aspiration and trephine biopsy were not done in view of skeletal involvement. Cerebrospinal fluid analysis was normal.

Histopathological examination of the cervical lymph node biopsy specimen showed B-cells with diffuse growth pattern and large lymphocytes, round in shape, pleomorphic nuclei, medium to large in size. The immunohistochemistry panel of the specimen showed positivity to B-cell antigens CD19, CD20, CD45, BCL-2 and negativity for CD3, CD5, CD10, MYC and BCL-6 and was diagnosed to have Diffuse Large B-Cell Lymphoma in December 2009. The proliferation rate was extremely high with numerous mitotic figures. Approximately 90% of cells expressed proliferation antigen Ki-67, which was recognized by the antibody MIB-1. So, overall staging evaluation revealed a stage IV Diffuse large B-cell lymphoma.

**Figure. 1:**
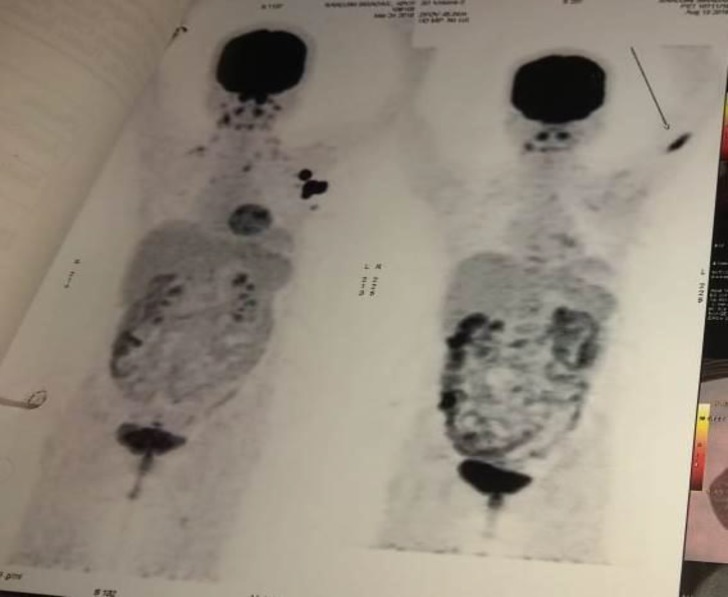
Showing Positron Emission Tomography- Computed Tomograohy (PET-CT) image of the patient with hyper metabolic left axillary lymph nodes.

The patient was managed with supportive care for tumor lysis syndrome prevention, and treatment was started with low dose COP [cyclophosphamide, oncovin (vincristine) and prednisolone] as reduction or prophase to prevent tumor lysis syndrome. Definitive treatment was done with 6 cycles of R-CHOP (Rituximab 375 mg /m2 D1, Cyclophosphamide 750 mg/m2 D1, Doxorubicin 50 mg /m2 D1, Vincristine 1,4 mg/ m2 and Prednisone 100mg per orally from D1-D5) till May 2010. Post- treatment PET-CT showed complete metabolic response and was under regular follow-up with the treating doctor. After 2 years, again she developed similar symptoms and managed with **1**^st^** Relapse: **July 2012→treated with R-DHAP (Rituximab 375 mg /m2 D1, Cisplatin 100 mg/ m2 D1, Cytarabine 2000mg/ m2 IV 12 hourly on D2, and Dexamethasone 40 mg D1-D4); completed in January 2013 and was under regular follow-up with the treating Doctor. After one and a half years, she again developed similar symptoms and managed with **2**^nd^ **Relapse: **August 2014 → treated with R-ICE (Rituximab 375 mg /m2 D1, Ifosfamide 5000mg/ m2 IV on D2, MESNA 5000mg/m2 IV in combination with Ifosfamide dose, Etoposide 100mg/m2 from D1-D3 and Carboplatin AUC 5 on D2, with G-CSF support for 4-cycles followed by allogenic HSCT) → completed in June 2015 and PET-CT showed a complete metabolic response. She was under regular follow-up for her disease recurrence. After one and a half years, she developed isolated axillary lymphadenopathy and managed with **3**^rd^** Relapse: **December 2016: isolated axillary lymphadenopathy (Figure. 1) → confirmed DLBCL by histopathology and immune histochemistry. PET-CT showed hyper metabolic regions in the left axillary regions (Figure 1).

This time, the patient was reluctant for any kind of chemotherapy. She was counselled about the new option of immunotherapeutic drug, Nivolumab. Nivolumab was administered with 3mg per kg every 2 weeks, and after 4 cycles she had a complete response which was confirmed with PET-CT. The patient tolerated immunotherapy very well without any side effects. Now, she is continuing the same dose and schedule, completed 12^th^ cycle last week, without any symptoms of the disease or any adverse reactions of the drug. It is planned for a total of 2 years of post-remission Nivolumab immunotherapy.

## Discussion

 Patients with relapsed or refractory aggressive B-cell Non-Hodgkin lymphoma (B-NHL) have a poor outcome. Lymphomas were one of the first cancers to show sensitivity to manipulations of the immune system. Nivolumab targeting the PD-1 receptor has shown promising results in several malignancies like melanoma, renal and lung carcinoma and Hodgkin lymphoma. Data for aggressive B-NHL are just emerging. The programmed death-1 (PD-1) pathway is an immune checkpoint to attenuate T-cell–mediated immune responses and may be exploited by tumors to avoid immune surveillance. Immune blockade of the PD-1/PD-L1 interaction by monoclonal antibodies can restore the antitumor activity of cytotoxic T cells ^2^_._

PD-1–blocking antibodies (nivolumab and pembrolizumab) produced durable objective responses and improved overall survival (OS) in patients with solid tumors and hematologic malignancies, including HL^3^. Nivolumab therapy resulted in ORRs of 36% and 40% among patients with DLBCL and FL, respectively. With continued nivolumab therapy, the depth of objective responses may improve as demonstrated by one patient with DLBCL with an initial PR (at 16 weeks) that converted to a CR (at 72 weeks) with extended treatment. Response durations exceeded 1 year for two (one each with FL and DLBCL) of three patients who achieved a CR and ≥ 6 months for patients with FL who achieved a PR^4^.

## CONCLUSION

 Cancer immunotherapy that harnesses the host immune system in novel ways to kill tumor cells is emerging. Immunotherapy offers promising opportunities with the potential to induce sustained remissions and is expected to become a “game changer” for the treatment of patients with cancer. Nivolumab was well-tolerated and exhibited antitumor activity in extensively pretreated patients with relapsed or refractory B- cell lymphomas. Additional studies of Nivolumab in these diseases are ongoing.
